# A nomogram for predicting feasibility of laparoscopic anterior resection with trans-rectal specimen extraction (NOSES) in patients with upper rectal cancer

**DOI:** 10.1186/s12893-021-01290-4

**Published:** 2021-06-17

**Authors:** Zhen-Yu Zhang, Zhe Zhu, Yuanyuan Zhang, Li Ni, Bing Lu

**Affiliations:** Department of General Surgery, Department of Colorectal Surgery, Shanghai East Hospital, School of Medicine, Tongji University, No. 150 Jimo Road, Pudong New District, Shanghai, 200120 China

**Keywords:** Rectal cancer, Laparoscopic anterior resection, Natural orifice specimen extraction surgery, Nomogram, Feasibility

## Abstract

**Background:**

Laparoscopic anterior resection with trans-rectal specimen extraction (NOSES) has been demonstrated as a safe and effective technique in appropriate patients with upper rectal cancer (RC). However, improper selection of RC candidates for NOSES may lead to potential surgical and oncological unsafety as well as complications such as bacteria contamination and anastomotic leak. Unfortunately, no tools are available for evaluating the risk and excluding improper cases before surgery. This study aims to estimate its clinical relevancy and to investigate independent clinical-pathological predictors for identifying candidates for NOSES in patients with upper RC and to develop a validated scoring nomogram to facilitate clinical decision making.

**Methods:**

The study was performed at Shanghai East hospital, a tertiary medical center and teaching hospital. 111 eligible patients with upper RC who underwent elective laparoscopic anterior resection between February and October of 2017 were included in the final analysis. Univariate and multivariate analyses were performed to compare characteristics between the two surgical techniques. Odds ratios (OR) were determined by logistic regression analyses to identify and quantify the clinical relevancy and ability of predictors for identifying NOSES candidate. The nomogram was constructed and characterized by c-index, calibration, bootstrapping validation, ROC curve analysis, and decision curve analysis.

**Results:**

Upper RC patients with successful NOSES tended to be featured with female gender, negative preoperative CEA/CA19-9, decreased mesorectum length (MRL), ratio of diameter (ROD) and ratio of area (ROA) values, while no significant statistical correlations were observed with age, body mass index (BMI), tumor location, and tumor-related biological characteristics (ie., vascular invasion, lymph node count, TNM stages). Furthermore, the two techniques exhibited comparably low incidence of perioperative complications and achieved similar functional results under the standard procedures. The nomogram incorporating three independent preoperative predictors including gender, CEA status and ROD showed a high c-index of 0.814 and considerable reliability, accuracy and clinical net benefit.

**Conclusions:**

NOSES for patients with upper RC is multifactorial; while it is a safe and efficient technique if used properly. The nomogram is useful for patient evaluation in the future.

**Supplementary Information:**

The online version contains supplementary material available at 10.1186/s12893-021-01290-4.

## Introduction

The notion of natural orifice specimen extraction surgery or NOSES for rectal cancer (RC) defines a modified procedure of specimen extraction through natural passages (i.e., rectum and vagina) and subsequent intracorporal digestive tract reconstruction in suitable patients with RC [1, 2]. The NOSES is increasingly implemented in upper RC and sigmoid cancer, which retrieves specimen through the distal rectum after a similar process of tumor resection and lymphadenectomy to conventional laparoscopy-assisted anterior resection (AR) but without additional abdominal wall incision [3–5]. NOSES has several distinct advantages as a result of its dedication for extremely minimal invasiveness based on a strict principle of contamination-free surgical manipulations [2, 6, 7]. Compared with laparoscopy-assisted surgery (Mini-Lapa), patients receiving NOSES tend to experience an increased rate of comfortability [8] and a comparable frequency of complications with a shortened time for recovery and hospital stay [7, 9–11]. However, improper application of NOSES in patients with upper RC may lead to potential surgical and oncological consequences and complications such as bacterial contamination and anastomotic bleeding and leak [2, 12, 13]. Unfortunately, current evaluation of RC candidates for NOSES is frequently left to the experience, expertise and discretion of surgeons [13], indicating a great space for improvement. Moreover, evidence-based consensuses on how to select proper patients remain unavailable [2, 13], which is becoming an obstacle for standardization and wide application of NOSES in upper RC.

In this study, we anticipated that many local parameters associated with surgical manipulations might be helpful in determination of NOSES in patients with upper RC. A retrospective study was performed with univariate and multivariate logistic analyses to identify potential predictors and a validated predictive nomogram were also developed to offer an accurate evaluation tool for auxiliary determination of NOSES with trans-rectal specimen extraction in patients with high RC.

## Materials and methods

### Patients and variables

**Fig. 1:**
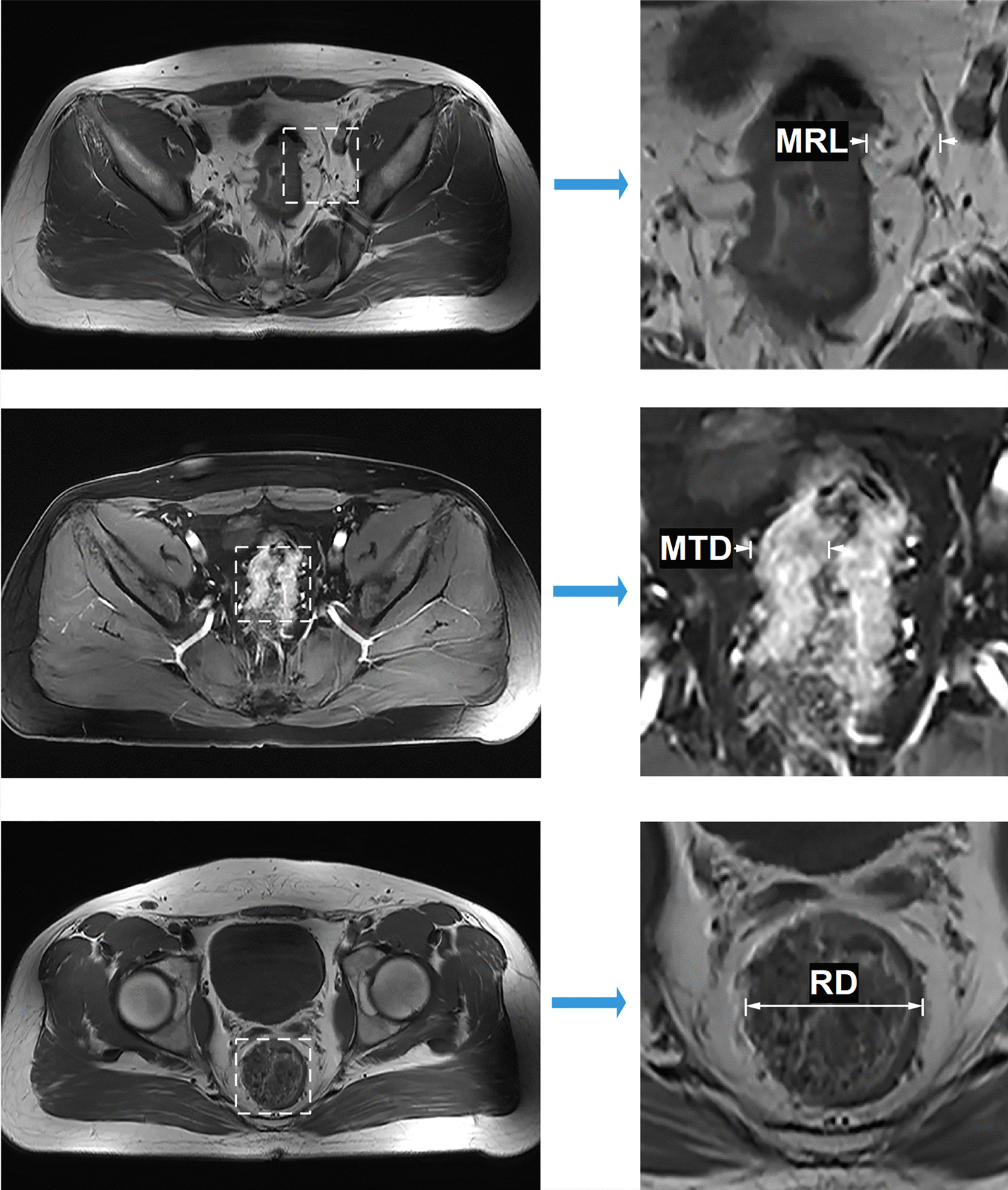
Schematics of the derivations of MRL, MTD and RD based on MRI

Data of consecutive patients (n = 138) with histologically proven upper RC (rectal cancer located above the peritoneal reflection) who received NOSES or Mini-Lapa at the department of colorectal surgery, Shanghai East Hospital, between February and October of 2017 were retrospectively extracted from our prospectively stored database. Patients were excluded with the following criteria: (1) history of pelvic/anal trauma/surgery/radio-chemotherapy (n = 13), (2) emergency surgery due to bleeding, bowel obstruction and perforation (n = 9), (3) multiple tumor masses (n = 4) and (4) concurrent anorectal stenosis (n = 1). Variables for patients analyzed included age, gender, BMI (< 25 or ≥ 25 kg/m^2^), preoperative CEA (> 5.2 ng/mL was considered positive), CA19-9 (> 27 U/mL was considered positive), tumor location (distance from anal verge to inferior margin of the tumor), MTD (maximum tumor cross-sectional diameter), MRL (mesorectum length), RD (rectum diameter), ROD (ratio of diameter), ROA (ratio of area) and tumor-related parameters such as differentiation, neural/vascular/lymphatic invasion, lymph node count (LNC), positive lymph node count (PLNC), pTNM and AJCC stages (8th edn). The values of RD, MRL, ROA and ROD were derived according to preoperative MRI scan of individual patients by experienced radiologists. Here, RD was defined as a maximum diameter of the rectum below the tumor location. MRL referred to the length of the mesorectum where the tumor had a maximum cross-sectional diameter. We assumed that the bilateral MRL values were similar. So, ROD was defined as (2*MRL + MTD)/RD. Accordingly, ROA was estimated as the summary of areas of the tumor and mesorectum divided by cross-sectional area of the rectum. Schematics of the derivations were shown below (Fig. 1). In addition, common perioperative complications and functional results associated with laparoscopic anterior resection were also analyzed.

### Algorithms and workflow for patients receiving surgery

The working algorithms was made for patients on the basis of surgical and oncological safety. Although all patients were initially considered candidates for NOSES, patients with apparent unfavorable features (such as a combinative or specific evaluation of male gender, very high BMI as well as very large tumor diameter in a case-by-case manner) were subjected to conventional Mini-Lapa directly. NOSES was tried in the patient without any known apparent unfavorable features. If the patient was demonstrated unsuccessful with NOSES, he/she would be subjected to a conversion to Mini-Lapa at last. Both cases with direct and conversion to Mini-Lapa were considered actual Mini-Lapa group subjects and were combined together before analysis.

**Fig. 2:**
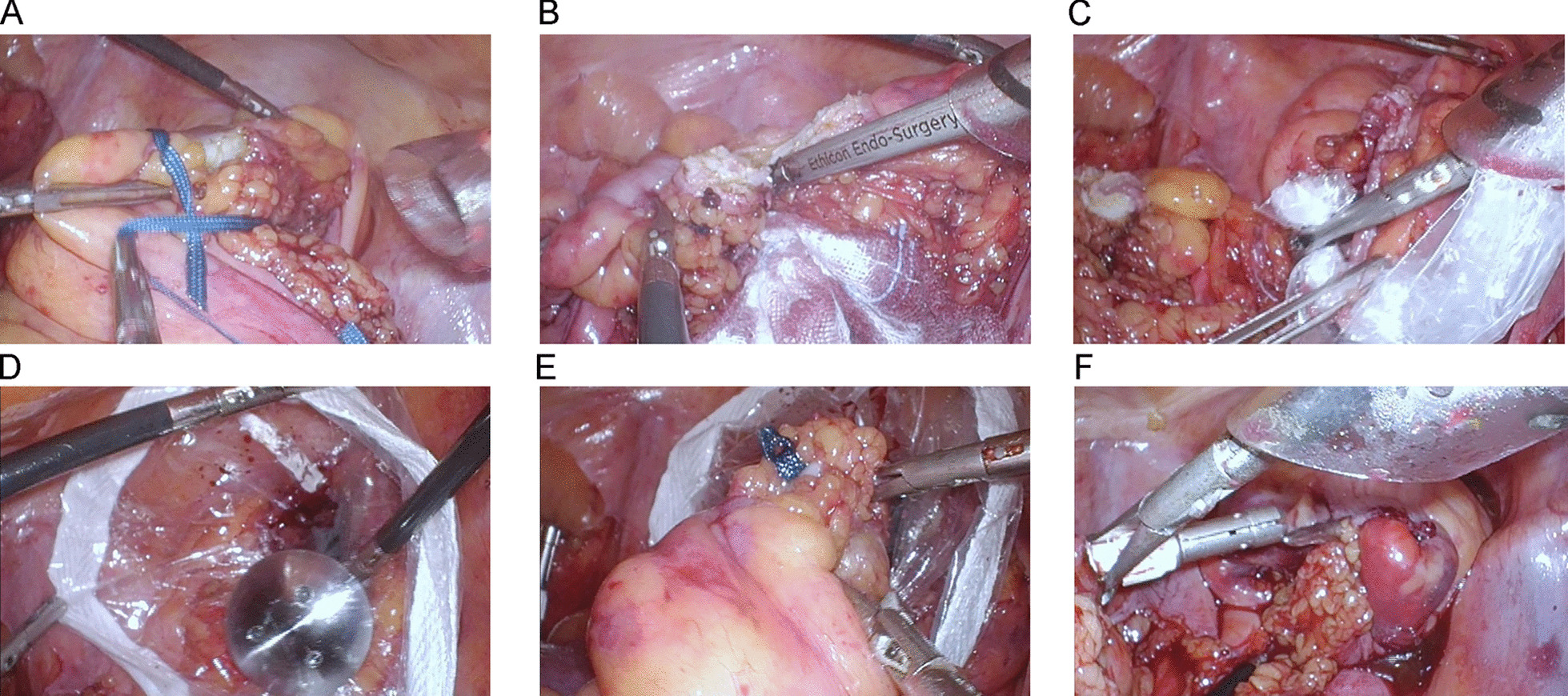
Procedures for laparoscopic anterior resection of upper RC with trans-rectal specimen extraction (NOSES). Laparoscopic surgeries were performed with general anesthesia and reverse Trendelenburg position was indicated. The standard surgical procedure for high RC mainly included high ligation of the inferior mesenteric artery, D3 lymphadenectomy, mobilization of sigmoid and rectum, dissection and retrieval of specimen and intracorporal anastomosis. As for laparoscopic anterior resection of high RC with trans-rectal specimen extraction (NOSES), proximal colon was dissected at approximately 10 cm away from the tumor followed by ligation of the distal rectum using silk ribbon (blue) at least 1 to 2 cm from the lower edge of the tumor (**A**) after being naked. The distal rectum was then dissected by ultrasound knife (**B**). Next, a plastic specimen protection bag was introduced into the pelvis through trocar at the right lower quadrant and trans-anal insertion of a Kocher’s clamp though the distal rectal stump was performed to grasp and pull the bottom half of the bag out of the body after four finger anal dilation (**C**). The anvil was placed in the cavity through the bag (**D**) and the specimen was dragged out thereafter (**E**). Intracorporal anastomosis with circular stapler (F)

Procedures for NOSES were generally similar to conventional laparoscopy-assisted AR, excepting for a serial modified way of trans-rectal extraction with a specific plastic specimen protector followed by bowel reconstruction by staplers [14]. A schematic (Fig. 2) explaining the key steps for specimen extraction and digestive tract reconstruction was shown below. Additionally, a small median incision of lower abdomen was used for extraction of specimen in patients who finally received Mini-Lapa.

### Statistical analysis

Continuous variables were presented as mean ± SD (standard deviation) or median ± IQR (interquartile range), and compared by independent *t* test or Mann-Withney *U* test, according to data distribution. Categorical factors were expressed as frequency (n) and proportion (%), and compared with Chi-squared test or Fisher exact test. Preoperative imaging factors were measured by experienced radiologists. Receiver operating characteristic (ROC) curve analyses were also applied to determine the predictive ability and optimized cutoff values of these measures by maximization of the Youden index. Univariate logistic regression analyses incorporating above-mentioned variables were used to calculate the association between clinical-pathological factors and NOSES with odds ratio (OR) as an effective measure for quantifying the strength of clinical relevancy and predictors. Only statistically significant preoperative variables were further included in the multivariate logistic regression analysis in a forward stepwise manner. A predictive nomogram was developed and characterized based on the final multivariate formula using our previously reported method [15]. All the analyses were processed with SPSS 17.0 (SPSS Inc.) and the R 3.3.3 program (https://www.r-project.org). A two-sided *P* value < 0.05 was considered statistically significant.

## Results

### ROC curve analysis and baseline characteristics

**Fig. 3:**
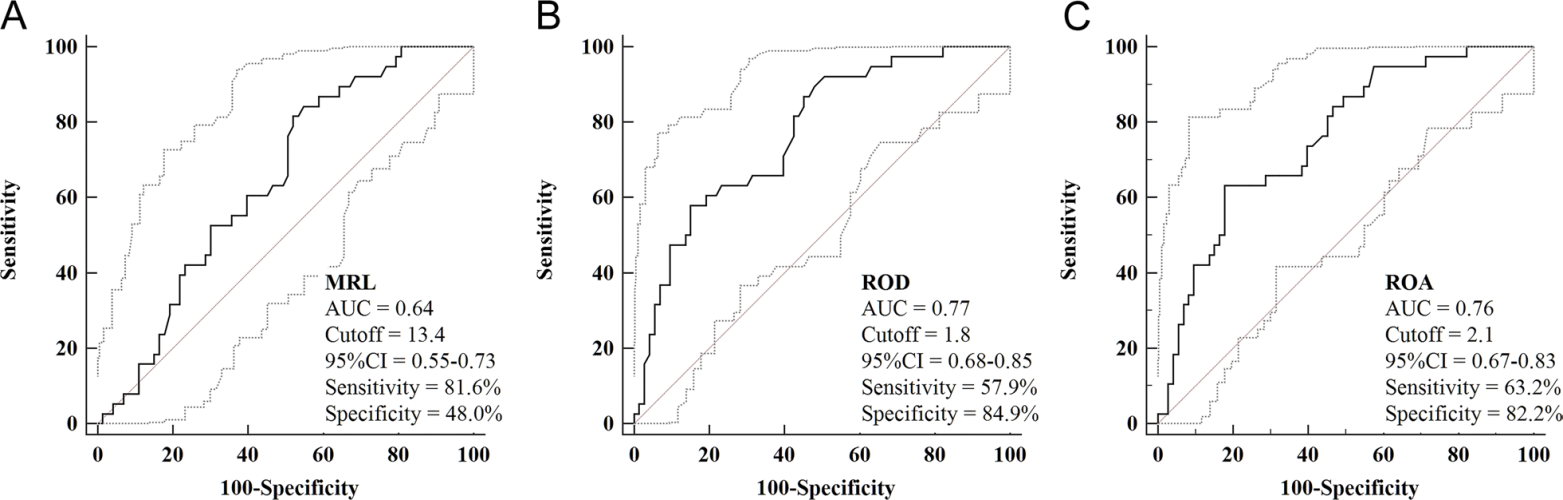
ROC curve analyses for MRL (**A**), ROD (**B**) and ROA (**C**)

A total of 111 eligible patients (NOSES = 73, Mini-Lapa = 38) were included in the final analysis. ROC curve analyses showed the optimized cutoff values for MRL, ROD and ROA were 13.4, 1.8 and 2.1 with an area under the curve (AUC) of 0.64, 0.77 and 0.76, respectively (Fig. 3).

Further comparison between the two groups showed that NOSES was more frequently performed in patients with a female gender, negative CEA/CA19-9 status and was associated with decreased MRL, ROD and ROA. No significant difference was seen in age, BMI, tumor location, differentiation, lymph-vascular invasion, LNC/PLNC and TNM stages (Table 1). Moreover, results of an additional sub-group analysis (Additional file 1: Table S1) indicated a very similar trend of variable correlations among direct Mini-Lapa, Mini-Lapa conversion and NOSES groups. Besides, the two techniques exhibited comparably low incidence of perioperative complications (i.e., surgical site infections, respiratory infection, urinary infection, anastomotic bleeding, anastomotic leak, urinary injury, urinary retention, postoperative intestinal obstruction, organ dysfunction, patient deaths within 30 days after surgery, anastomotic stricture and development of local recurrence during follow-up) and achieved similar functional results (i.e., urinary/sexual dysfunction and incontinence) under the standard procedures (Additional file 1: Table S1). Finally, Post-Hoc multiple comparisons clarified that the Mini-Lapa group and Mini-Lapa conversion group represented its own characteristics and also common features which were both useful in being discriminated from the patients receiving NOSES (Additional file 1: Table S1).

**Table 1:** Clinicopathological characteristics between NOSES and laparoscopy-assisted surgery (Mini-Lapa)

Factors	NOSES		Mini-Lapa		*P* value
	Mean (n)	SD (%)	Mean (n)	SD (%)	
Gender (F/M, n/%)	34/39	46.6/53.4	6/32	15.8/84.2	0.001
Age (mean ± SD, year)	60.3	11.8	64.8	10.3	0.050
BMI (< / ≥ 25)	59/14	80.8/19.2	29/9	76.3/23.7	0.578
CEA (−/ +)	63/10	86.3/13.7	20/18	52.6/47.4	< 0.001
CA19-9 (−/ +)	66/7	90.4/9.6	27/11	71.1/28.9	0.009
Tumor location (mean ± sd, cm)	9.3	3.5	10.3	2.8	0.130
MTD (mean ± sd, mm)	14.3	6.7	17.1	9.5	0.072
RD (mean ± sd, mm)	27.3	6.8	31.3	10.4	0.068
MRL (< / ≥ 13.4)	35/38	47.9/52.1	7/31	18.4/81.6	0.002
ROD (< / ≥ 1.8)	62/11	84.9/15.1	17/21	44.7/55.3	< 0.001
ROA (< / ≥ 2.1)	60/13	82.2/17.8	14/24	36.8/63.2	< 0.001
Differentiation (G1 + G2/G3 + G4)	58/15	79.5/20.5	28/10	73.7/26.3	0.490
Neural invasion (−/ +)	61/12	83.6/16.4	27/11	71.1/28.9	0.123
Vascular invasion (−/ +)	54/19	74.0/26.0	31/7	81.6/18.4	0.369
Lymphatic invasion (−/ +)	53/20	72.6/27.4	21/17	55.3/44.7	0.066
LNC (mean ± SD)	13.8	4.6	15.6	5.0	0.051
PLNC (mean ± SD)	1.7	3.2	1.8	3.1	0.871
T stage (T1/2/3/4)	11/17/34/11	15.1/23.3/46.6/15.1	2/9/22/5	5.3/23.7/57.9/13.2	0.432
N stage (N0/1/2)	44/15/14	60.3/20.5/19.2	21/11/6	55.3/28.9/15.8	0.601
M stage (M0/1)	69/4	94.5/5.5	34/4	89.5/10.5	0.329
TNM stage (Stage I/II/III/IV)	21/21/27/4	28.8/28.8/37.0/5.5	7/14/13/4	18.4/36.8/34.2/10.5	0.473

BMI, body mass index; MTD, maximum tumor cross-sectional diameter; RD, rectum diameter; MRL, mesorectum length; ROD, ratio of diameter; ROA, ratio of area

### Univariate and multivariate logistic regression analyses

**Fig. 4:**
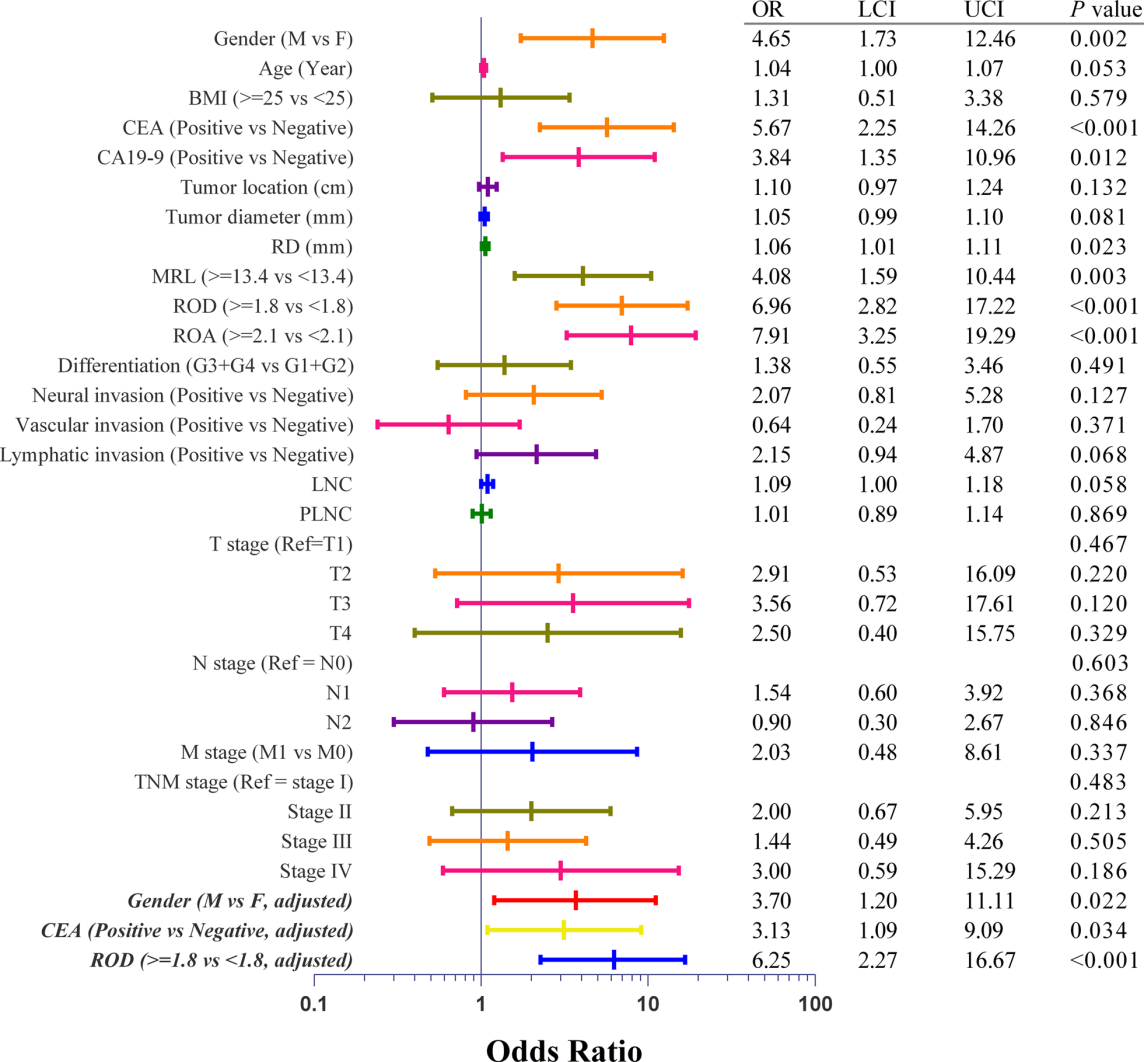
Forest plots for univariate and multivariate logistic analyses

Univariate logistic regression analyses were conducted to explore the potential of clinical-pathological variables as a favorable indicator for NOSES. The results (Fig. 4) were consistent to those in above-mentioned clinical-pathological comparisons. Preoperative variables which were statistically significant in the univariate analysis were then incorporated in a stepwise multivariate logistic regression analysis to yield independent predictors for NOSES. Since ROC curve analyses demonstrated that ROD exhibited a similar predictive ability but was simplified in comparison with ROA, we included ROD instead of ROA in the multivariate analysis. The results showed that NOSES were more likely to be conducted in female, CEA negative and lower ROD patients (Fig. 4).

### Development of a nomogram for NOSES

**Fig. 5:**
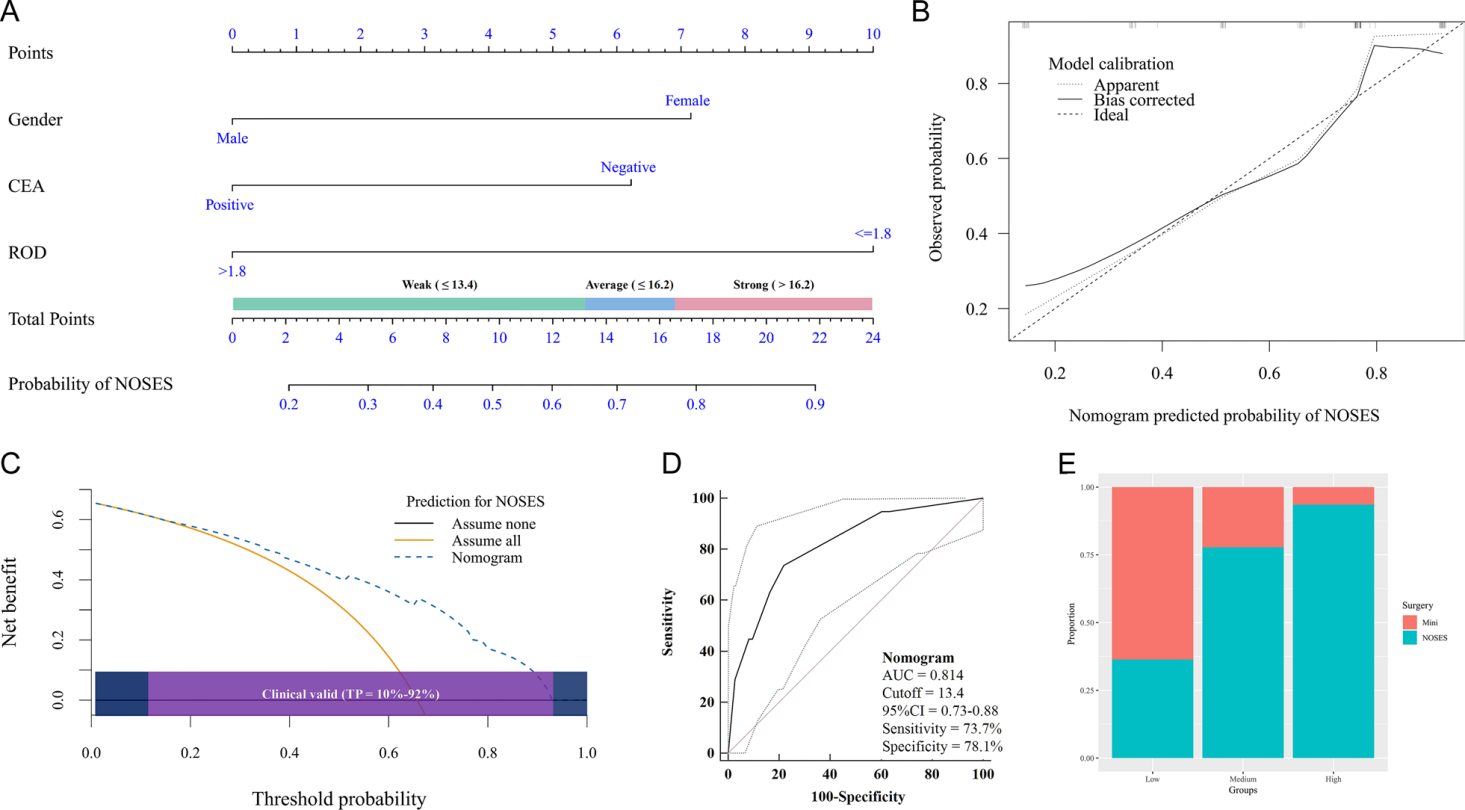
Development and validation of the nomogram for selection of RC patients for NOSES. **A** the nomogram; **B** calibration plot; **C** decision curve analysis with a wide threshold probability (TP); **D** ROC curve analysis; **E** stacked column chart

Based on the final covariate formula of multivariate logistic regression analysis, we developed a nomogram to predict the possibility of NOSES before surgery. In brief, the nomogram including patient gender, CEA status and ROD level showed a considerable discrimination with a c-index of 0.814 (Fig. 5A).

### Internal validation of the nomogram

The discrimination remained stable (c-index = 0.800) after 1000 resample bootstrapping validation to correct overfitting. Calibration by 1000 resample bootstrapping implied a good agreement between nomogram predicted and observed possibility of NOSES (Fig. 5B). Decision curve analysis suggested the nomogram were associated with considerable net benefit and was clinical valid within probability thresholds between 10 and 92% (Fig. 5C). Moreover, the AUC (Fig. 5D) of nomogram to predict NOSES before surgery was 0.81 (95% CI 0.73–0.88), as estimated by the ROC curve analysis using nomogram-derived total points of patients as a predictive variable. Additionally, using the nomogram-derived score, we calculated the total points for individual patients and divided the patients into three subgroups of ascending possibility by tertiles (total points ≤ 13.4, 13.5–16.2 and > 16.2) to classify low, medium and high-grade preoperative recommendations for NOSES. Results showed that a preoperative higher total point was consistently associated with a significantly increased proportion of patients who received NOSES (Fig. 5E, P < 0.001).

## Discussion

As a bridge between NOTES and conventional laparoscopy, NOSES for upper RC has exhibited comparable technical safety and oncological outcomes but more dedicated intention and improved results for extremely minimal invasiveness. This was confirmed by high quality evidence from randomized controlled trail and meta-analysis [10, 11]. Excepting for requirements for expertise of surgeons and surgical equipment, a proper patient identifying model and evaluation tool is fundamental to ensure the security and effectiveness of the technique [13, 16]. In the light of above considerations, we performed the study and there were some new findings in addition to reproducing some known correlators.

Generally, upper RC patients with successful trans-rectal specimen extraction tended to be featured with female gender, negative CEA/CA19-9, decreased MRL, ROD and ROA values, while no significant associations were seen with age, BMI, tumor location, and tumor-related biological characteristics (ie., vascular invasion, LNC, TNM stages). More importantly, univariate and multivariate analyses identified some novel factors reflecting and deciding the degree of difficulty in retrieval of the specimen through the distal rectum. Firstly, an elevated CEA often implies an augmented tumor volume and deepened bowel wall penetration [17]. Thus, a negative CEA may indicate less difficulty in tumor extraction and fewer chances of contaminations. Considering preoperative CEA assay is a simple and routine test for patients with RC, it may also be a new marker for identifying candidates of NOSES. Secondly, the fact that a higher proportion of female patients receiving NOSES was partially a result of their widened pelvis [18] which better facilitated surgical manipulations. However, which parameters for pelvic magnitudes play a major role remains to be clarified. Thirdly, we demonstrated that a decreased ROD was in favorable of the performance of NOSES. The results were consistent with our initial proposal of ROD which was based on the assumption that the distal mesorectum and rectal wall had a certain degree of elasticity (roughly estimated as RD) to allow for a safe and smooth pull-through of the resected specimen and the procedure could be more likely restricted or interrupted where the specimen had a largest diameter (estimated as [2*MRL + MTD]). It should be noted that the length of specimen varied depending on numerous conditions and was unlikely be to a limiting factor for trans-rectal specimen extraction. The situation was similar for maximum tumor diameter as a less accurate predictor since a majority of RCs were infiltrative and not necessarily correlated with MTD. Furthermore, although the ROA was superior to the ROD as indicated by ROC curve analysis, we did not incorporate it into final analysis because the derivation was relatively complicated which might obstacle its clinical application. In addition, ROC curve analysis also suggested that MRL was more sensitive while ROD and ROA were more specific in identifying patients suitable for NOSES. Combinative use of them might lead to increased predictive ability, just like the nomogram. Fourthly, our results also showed that pathological characteristics (such as differentiation, LNC, PLNC and TNM stages) were similar irrespective of the techniques performed. This was also in accordance with the knowledge that NOSES for RC shares a common surgical mobilization and lymphadenectomy [19] and exerts no significant influences on stage migration or decision of postoperative therapies; thus, rendering a similar clinical outcome to Mini-Lapa [19, 20]. This was also consistent with the sub-group analysis which suggested comparably low incidence of perioperative complications and similar functional results under the standard procedures for them. We did not observe significant difference in BMI between the two groups; nevertheless, it only meant that BMI was less important compared with other regional factors. Other explains might be that most of RC patients experienced a certain extent of weight loss due to tumor exhaustion and many of our patients had a BMI of less than 25 when firstly diagnosed. In addition, the results of Post-Hoc analyses implied some same (such as gender and CEA) as well as different (such as ROD) characteristics between Mini-Lapa group and Mini-Lapa conversion groups which were important identifiers distinctive from the NOSES groups. It also suggested that consideration of them as a group could be more practical and less unlikely to losing discriminative efficiency for the multivariate predictive model.

Lastly, we developed a nomogram for NOSES with graphical representation of a newly constructed multivariate predictive model. This nomogram achieved a c-index of 0.814 and considerable reliability, accuracy and net benefit. The nomogram will be helpful in excluding unsuitable cases in order to prevent potential adverse events of NOSES. This tool is also useful in identifying suitable cases for NOSES and improvement of informative decision making. Additionally, it also can be used to select homogeneous patients for NOSES-related clinical trials.

Our study had some limitations that have persuaded us to interpret with caution. Our study was a retrospective study and the patient sample was relatively small, despite that all study data were prospectively collected and stored; the influence of selection bias might be underestimated, although multivariate analysis and internal validation were applied. The nomogram was an uncertainty in itself [21]; hence external validation study was further needed to repeat the ability and results for the nomogram.

In summary, the results of our study are in keeping with the conclusion that feasibility of NOSES with trans-rectal specimen retrieval for patient with upper RC is multifactorial. The factors such as patient gender, CEA level, and ROD derived from preoperative imaging are demonstrated to be relatively more important predictors, offering new criteria and tools for patient selection for NOSES. Moreover, the nomogram based on these covariates has shown considerable discrimination, reliability, reproducibility and clinical net benefit, allowing for potential clinical application in the future. Additionally, our study can be extended to incorporate pelvis-related factors to improve the efficiency of the nomogram in future studies.

## Supplementary Information

**Additional file 1: Table S1.** Clinicopathological characteristics, perioperative complications and functional results among groups of NOSES, direct laparoscopy-assisted surgery (Mini-Lapa) and laparoscopic conversions.

## Data Availability

The datasets used and analysed during the current study available from the corresponding author on reasonable request.
