# Supplementary material for: A nomogram for predicting feasibility of laparoscopic anterior resection with trans-rectal specimen extraction (NOSES) in patients with upper rectal cancer
Source: BMC Surg. 2021 Jun 17;21:296. doi: 10.1186/s12893-021-01290-4 (PMC8212478; doi:10.1186/s12893-021-01290-4)
Supplement: Supplementary file 1 — Additional file 1: Table S1. Clinicopathological characteristics, perioperative complications and functional results among groups of NOSES, direct laparoscopy-assisted surgery (Mini-Lapa) and laparoscopic conversions. [file 12893_2021_1290_MOESM1_ESM.docx]

**Supplemental table 1** Clinicopathological characteristics, perioperative complications and functional results among groups of NOSES, direct laparoscopy-assisted surgery (Mini-Lapa) and laparoscopic conversions.

| ***Factors*** |  | **NOSES (n=73)** | |  | **Direct mini-laparoscopy (n=12)** | |  | **Mini-laparoscopy conversion (n=26)** | |  | ***P* for trend** | ***P* value of post hoc analysis** | | | | |
| --- | --- | --- | --- | --- | --- | --- | --- | --- | --- | --- | --- | --- | --- | --- | --- | --- |
|  |  | **mean (n)** | **SD (%)** |  | **mean (n)** | **SD (%)** |  | **mean (n)** | **SD (%)** |  |  | P1 |  | P2 |  | P3 |
| **Gender (F/M, n/%)** |  | 34/39 | 46.6/53.4 |  | 2/12 | 16.7/83.3 |  | 4/22 | 15.4/84.6 |  | 0.006 | >0.05 |  | <0.05 |  | >0.05 |
| **Age (mean ± SD, year)** |  | 60.3 | 11.8 |  | 64.6 | 9.0 |  | 64.8 | 11.0 |  | 0.147 | 0.223 |  | 0.082 |  | 0.956 |
| **BMI (</≥25)** |  | 59/14 | 80.8/19.2 |  | 6/6 | 50.0/50.0 |  | 23/3 | 88.5/11.5 |  | 0.021 | >0.05 |  | >0.05 |  | <0.05 |
| **CEA (-/+)** |  | 63/10 | 86.3/13.7 |  | 5/7 | 41.7/58.3 |  | 15/11 | 57.7/42.3 |  | <0.001 | <0.05 |  | <0.05 |  | >0.05 |
| **CA19-9 (-/+)** |  | 66/7 | 90.4/9.6 |  | 8/4 | 67.7/33.3 |  | 19/7 | 73.1/26.9 |  | 0.028 | >0.05 |  | >0.05 |  | >0.05 |
| **Tumor location (mean ± sd, cm)** |  | 9.3 | 3.5 |  | 10.8 | 2.7 |  | 10.1 | 2.9 |  | 0.274 | 0.164 |  | 0.289 |  | 0.518 |
| **MTD (mean ± sd, mm)** |  | 14.3 | 6.7 |  | 22.0 | 13.7 |  | 14.9 | 5.9 |  | 0.140 | 0.229 |  | 0.964 |  | 0.299 |
| **RD (mean ± sd, mm)** |  | 27.3 | 6.8 |  | 36.0 | 12.1 |  | 29.1 | 9.0 |  | 0.049 | 0.094 |  | 0.749 |  | 0.264 |
| **MRL (</≥13.4)** |  | 35/38 | 47.9/52.1 |  | 1/11 | 8.3/91.7 |  | 6/20 | 23.1/76.9 |  | 0.007 | <0.05 |  | >0.05 |  | >0.05 |
| **ROD (</≥1.8)** |  | 62/11 | 84.9/15.1 |  | 1/11 | 8.3/91.7 |  | 16/10 | 61.5/38.5 |  | <0.001 | <0.05 |  | <0.05 |  | <0.05 |
| **ROA (</≥2.1)** |  | 60/13 | 82.2/17.8 |  | 1/11 | 8.3/91.7 |  | 13/13 | 50.0/50.0 |  | <0.001 | <0.05 |  | <0.05 |  | <0.05 |
| **Differentiation (G1+G2/G3+G4)** |  | 58/15 | 79.5/20.5 |  | 8/4 | 67.7/33.3 |  | 20/6 | 76.9/23.1 |  | 0.615 | >0.05 |  | >0.05 |  | >0.05 |
| **Neural invasion (-/+)** |  | 61/12 | 83.6/16.4 |  | 7/5 | 58.3/41.7 |  | 20/6 | 76.9/23.1 |  | 0.128 | >0.05 |  | >0.05 |  | >0.05 |
| **Vascular invasion (-/+)** |  | 54/19 | 74.0/26.0 |  | 10/2 | 83.3/16.7 |  | 21/5 | 80.8/19.2 |  | 0.658 | >0.05 |  | >0.05 |  | >0.05 |
| **Lymphatic invasion (-/+)** |  | 53/20 | 72.6/27.4 |  | 7/5 | 58.3/41.7 |  | 14/12 | 53.8/46.2 |  | 0.178 | >0.05 |  | >0.05 |  | >0.05 |
| **LNC (mean ± SD)** |  | 13.8 | 4.6 |  | 15.1 | 4.1 |  | 15.9 | 5.4 |  | 0.135 | 0.374 |  | 0.054 |  | 0.623 |
| **PLNC (mean ± SD)** |  | 1.7 | 3.2 |  | 1.3 | 2.3 |  | 2.0 | 3.5 |  | 0.770 | 0.664 |  | 0.630 |  | 0.483 |
| **T stage (T1/2/3/4)** |  | 11/17/34/11 | 15.1/23.3/46.6/15.1 |  | 1/3/6/2 | 8.3/25.0/50.0/16.7 |  | 1/6/16/3 | 3.8/23.1/61.5/11.5 |  | 0.719 | >0.05 |  | >0.05 |  | >0.05 |
| **N stage (N0/1/2)** |  | 44/15/14 | 60.3/20.5/19.2 |  | 7/4/1 | 58.3/33.3/8.3 |  | 14/7/5 | 53.8/26.9/19.2 |  | 0.766 | >0.05 |  | >0.05 |  | >0.05 |
| **M stage (M0/1)** |  | 69/4 | 94.5/5.5 |  | 11/1 | 91.7/8.3 |  | 23/3 | 88.5/11.5 |  | 0.607 | >0.05 |  | >0.05 |  | >0.05 |
| **TNM stage (Stage I/II/III/IV)** |  | 21/21/27/4 | 28.8/28.8/37.0/5.5 |  | 3/4/4/1 | 25.0/33.3/33.3/8.3 |  | 4/10/9/3 | 15.4/38.5/34.6/11.5 |  | 0.802 | >0.05 |  | >0.05 |  | >0.05 |
| ***Perioperative complications and functional results*** |  |  |  |  |  |  |  |  |  |  |  |  |  |  |  |  |
| **Surgical site infections (Incision & organ space, No/yes, n)** |  | 68/5 | 93.2/6.8 |  | 11/1 | 91.7/8.3 |  | 25/1 | 96.2/3.8 |  | 0.811 | >0.05 |  | >0.05 |  | >0.05 |
| **Anastomotic bleeding (No/yes, n)** |  | 73/0 | 100.0/0 |  | 12/0 | 100.0/0 |  | 26/0 | 100.0/0 |  | / | / |  | / |  | / |
| **Anastomotic leak (No/yes, n)** |  | 73/0 | 100.0/0 |  | 12/0 | 100.0/0 |  | 26/0 | 100.0/0 |  | / | / |  | / |  | / |
| **Respiratory infection (No/yes, n)** |  | 69/4 | 94.5/5.5 |  | 11/1 | 91.7/8.3 |  | 25/1 | 96.2/3.8 |  | 0.856 | >0.05 |  | >0.05 |  | >0.05 |
| **Urinary injury (No/yes, n)** |  | 73/0 | 100.0/0 |  | 12/0 | 100.0/0 |  | 26/0 | 100.0/0 |  | / | / |  | / |  | / |
| **Urinary infection (No/yes, n)** |  | 68/5 | 93.2/6.8 |  | 10/2 | 88.3/16.7 |  | 24/2 | 92.3/7.7 |  | 0.578 | >0.05 |  | >0.05 |  | >0.05 |
| **Urinary retention (No/yes, n)** |  | 71/2 | 97.3/2.7 |  | 12/0 | 100.0/0 |  | 26/0 | 100.0/0 |  | 0.428 | >0.05 |  | >0.05 |  | >0.05 |
| **Postoperative intestinal obstruction (No/yes, n)** |  | 71/2 | 97.3/2.7 |  | 11/1 | 91.7/8.3 |  | 26/0 | 100.0/0 |  | 0.306 | >0.05 |  | >0.05 |  | >0.05 |
| **Organ dysfunction (No/yes, n)** |  | 73/0 | 100.0/0 |  | 12/0 | 100.0/0 |  | 26/0 | 100.0/0 |  | / | / |  | / |  | / |
| **Patient deaths within 30 days after surgery (No/yes, n)** |  | 73/0 | 100.0/0 |  | 12/0 | 100.0/0 |  | 26/0 | 100.0/0 |  | / | / |  | / |  | / |
| **Urinary dysfunction (No/yes, n)** |  | 73/0 | 100.0/0 |  | 12/0 | 100.0/0 |  | 26/0 | 100.0/0 |  | / | / |  | / |  | / |
| **Sexual dysfunction (No/yes, n)** |  | 73/0 | 100.0/0 |  | 12/0 | 100.0/0 |  | 26/0 | 100.0/0 |  | / | / |  | / |  | / |
| **Anastomotic stricture (No/yes, n)** |  | 73/0 | 100.0/0 |  | 12/0 | 100.0/0 |  | 26/0 | 100.0/0 |  | / | / |  | / |  | / |
| **Incontinence (No/yes, n)** |  | 73/0 | 100.0/0 |  | 12/0 | 100.0/0 |  | 26/0 | 100.0/0 |  | / | / |  | / |  | / |
| **Development of local recurrence during 3-year follow-up (No/yes, n)** |  | 73/0 | 100.0/0 |  | 11/1 | 91.7/8.3 |  | 26/0 | 100.0/0 |  | 0.104 | <0.05 |  | >0.05 |  | >0.05 |

Note: Statistical analyses were achieved by using One-way Anova or Kruskcal Wallis H test for continuous variables based on data distribution, and χ^2^ tests were used for categorical variables. Post hoc analyses were accomplished by using LSD/Tamhane's T2 or Bonferroni method after tests for trend. P1, P2 and P3 were post hoc multiple comparison p values between NOSES vs. direct Mini-Lapa, NOSES vs. Mini-Lapa conversion, and direct Mini-Lapa vs. Mini-Lapa conversion, respectively. All p values were two-sided.

BMI, body mass index; MTD, maximum tumor cross-sectional diameter; RD, rectum diameter; MRL, mesorectum length; ROD, ratio of diameter; ROA, ratio of area.
